# Supplementary material for: Are carbon emissions trading and green financial instruments synergistic? -Comprehensive quantitative research based on content analysis
Source: PLoS One. 2024 Mar 7;19(3):e0298601. doi: 10.1371/journal.pone.0298601 (PMC10919601; doi:10.1371/journal.pone.0298601)
Supplement: S1 Data — (ZIP) [file pone.0298601.s001.zip › Data/地级市Z_F工作报告.zip!/║╙─╧/║╫▒┌╩╨/└┤╫╘╣1⁄2╓┌║┼í╛┬φ┐╦╩2╛▌═°í┐_2015.docx]

各位代表：

　　现在，我代表市人民政府，向大会报告工作，请予审议，并请各位政协委员和其他列席人员提出意见。

　　一、2014年工作回顾

　　过去的一年，是我市经济社会发展中具有重要意义的一年。面对严峻复杂的经济形势，新一届市政府班子团结带领广大干部群众，全面落实省委、省政府和市委的决策部署，统筹推进改革发展稳定各项工作，较好完成了市十届人大一次会议确定的各项任务。初步核算，全市生产总值完成682亿元，比上年增长10.1%、增速居全省第1位；规模以上工业增加值完成429亿元，增长12.1%、居全省第4位；固定资产投资完成588亿元，增长20.8%、居全省第1位；公共财政预算收入完成47.1亿元，增长18.8%、居全省第3位；社会消费品零售总额完成161亿元，增长12.6%；预计城镇居民人均可支配收入23351元、农民人均纯收入11775元，分别增长10%和11%左右；主要经济指标增速位次前移，发展速度与产业结构、质量效益趋于协调，良好的发展成效使我市率先全面建成小康社会的基础更加坚实、支撑更加有力、前景更加广阔。

　　一年来，我们主要抓了以下几个方面的工作：

　　（一）突出做好三项重点工作，增长动力保持强劲。项目建设方面，270个市重点项目完成投资438亿元、完成年度计划的102%，其中新开工项目170个、竣工投产项目59个；27个省重点项目完成投资155亿元，完成年度计划的145.6%、完成计划比例居全省第1位。招商引资方面，引进省外资金240亿元，增长17.3%、居全省第2位；实际利用外资6.7亿美元，增长19.7%、居全省第4位；进出口总额3.2亿美元，增长22%、居全省第5位。企业服务方面，帮助企业协调解决问题197个，规模以上企业正常开工率超过95%，3家企业入选省百强百高企业，1家公司在新三板挂牌。金融机构存贷款余额分别达到477.7亿元和438.3亿元、分别增长12.1%和12.5%，存贷比91.6%、居全省第1位，争取统贷统还资金23.3亿元，成功发行企业债券9亿元；挖潜盘活用地指标1.5万亩，报批各类建设用地9626亩、供地7723亩；完成各类技能培训7.3万人次。4个产业集聚区和3个园区完成固定资产投资401亿元，主营业务收入突破1000亿元；商务中心区和4个特色商业区在建千万元以上项目40个；海关和出入境检验检疫局建成投用。成功创建全省首个全国质量强市示范城市，被确定为全国首家国土空间优化发展实验区。

　　（二）积极统筹产业协调发展，转型升级取得成效。3个主导产业、2个先导产业增加值分别增长13.4%和81.8%，占规模以上工业增加值的76.8%，其中高新技术产业增加值增长22.3%，产业结构趋于优化。休闲旅游和新型物流两个新业态快速发展，接待国内外游客和旅游综合收入分别增长16.2%和21.5%；新型物流业增加值占第三产业的比重逐步提高；房地产市场持续健康发展，第三产业增加值增长8%。建成高标准粮田18.4万亩，粮食总产实现“十一连增”、夏粮实现“十二连增”，农业科技和信息化水平进一步提升，国家农业综合开发现代农业园区、国家农业科技园区、国家信息化示范基地建设稳步推进，成功创建整市建制国家现代农业示范区，获批创建全国农业社会化服务综合标准化示范市，被确定为国家农业信息化进村入户试点市。

　　（三）科学推进新型城镇化，城乡功能逐步完善。预计城镇化率达到54.1%。城乡一体化试点市持续探索推进，示范区建设步伐加快，淇水湾商务休闲区6个主骨架项目开工，道路、管网等基础设施基本建成，淇河综合治理工程效果显现。淇滨区建成区提升和老城区十大建设工程分别完成投资10.7亿元、52.8亿元，老城区新增供热覆盖面积140万平方米，鹤山区首次实现集中供暖，浚县、淇县老城改造和新城建设统筹推进，干部移动学习云平台鹤壁分中心和鹤壁城市云计算中心建成投用。6个美丽乡村建设项目列为国家、省试点，新增7个传统村落列入国家级、省级保护名录，农村人居环境整治取得阶段性成效。晋豫鲁铁路建成通车，鹤辉高速、大海线施工进展顺利，鹤浚快速通道、东大线开工建设，市公路运输综合客运枢纽站建成启用、开通省内外客运班线35条，建设农村公路82.5公里。

　　（四）加快建设生态文明，环境品牌更加响亮。深入推进绿色低碳循环发展，完成造林绿化24.5万亩，森林覆盖率、城区绿化覆盖率分别达到31.7%和40.6%、均居全省前列，成功创建国家森林城市、省级园林城市。淇河湿地公园、盘石头水库生态环保等一批重点生态项目顺利实施，淇河水质在全省排名第一、常年水质保持二类以上。南水北调中线一期工程建成通水。围绕创建国家循环经济示范市，加快建设示范园区、示范企业，循环经济项目14个竣工、15个开工，完成投资14.6亿元，预计万元工业增加值能耗下降10%左右，成功创建全省唯一的国家节能减排财政政策综合示范城市。扎实开展蓝天、碧水和乡村清洁“三大工程”，城市环境空气优良率按新标准超过60%、居全省第1位，城市集中饮用水源地水质达标率100%，河流出境断面水质达标率保持省先进水平。

　　（五）全面深化改革创新，发展活力不断增强。政府职能转变和机构改革基本完成，行政审批项目精简78.2%、审批时限压缩75.4%，被确定为省行政审批标准化试点市。研究出台了支持鹤山区、山城区、开发区和城乡一体化示范区加快改革发展的系列政策措施，组建了公共资源交易中心、中小企业服务中心和文化旅游集团、镁业集团，工商登记制度改革取得明显成效。宝山产业集聚区被确定为省经济开发区。国家镁及镁合金质检中心通过考核验收，国家镁合金材料工程技术研究中心鹤壁中心、中科院半导体研究所河南研究院、南京林业大学家具设计与工程学博士后流动站（河南聆海）工作点挂牌成立，技术创新战略联盟实现突破，与郑州大学、河南工业大学等院校深度合作，与河南农业大学、河南财经政法大学、河南师范大学、河南省科学院等签订战略合作协议，新建市以上工程技术研究中心30家，实施重大科技专项6项。

　　（六）着力保障改善民生，人民生活持续提高。全市民生相关支出占公共财政预算支出的80.3%，新开工保障性住房15632套、基本建成16010套，20个新型社区达到入住条件，改造农村危房4826户，解决6.3万农村居民和师生饮水安全问题，实施了13个扶贫开发整村推进项目，盘石头水库三期移民搬迁正式启动。新增城镇就业4万人、农村劳动力转移就业2.2万人。居民健康档案建档率96.9%、新农合参合率99.6%，新农合大病保险二次补偿启动，新农合和城镇居民医保财政补助标准、人均基本公共卫生服务标准分别提高到320元、35元，企业退休人员养老金增长12.8%，社会保险覆盖面进一步扩大，城乡低保实现动态管理下的应保尽保。学前教育三年入学率达到83.7%、九年义务教育巩固率98.8%，改造了8所农村义务教育薄弱学校，13个市区中小学和幼儿园项目已有4个投入使用，鹤壁职业技术学院完成搬迁，鹤壁能源化工职业学院获批设立。“芝麻官大舞台”每周演出1次，与河南歌舞演艺集团合作每月演出1次，举办各类文化演出1000余场，成功举办第六届民俗文化节和首届文博会，淇河被命名为“中国诗河”，卫河（永济渠）浚县段、黎阳仓遗址入选世界文化遗产名录。新建农民体育健身场所156处，全民健身活动中心暨体育公园启动建设。被确定为全国创建幸福家庭活动试点市、全省人口均衡型社会综合配套改革示范市。公众法治环境满意度连续3年居全省第1位、安全感指数居全省第1位，被确定为全国法治城市创建活动先进单位。

　　一年来，我们切实加强民主法治建设，自觉接受人大法律和工作监督、政协民主监督、社会舆论监督，办理人大代表建议和政协提案306件、办结率100%。切实加强政府自身建设，从严从实开展党的群众路线教育实践活动，着力解决了46个群众反映强烈的突出问题，三个环节群众满意率均超过98%；深入推进依法行政，认真落实党风廉政建设责任制，强化行政、司法、监察、审计等监督，深入推进反腐倡廉。切实加强精神文明建设，开展了系列公民道德建设工程和文明创建活动，涌现出李应喜、张慧、彭兵先、唐全合等一批全国先进典型。切实加强国防教育、国防后备力量及人民防空建设，推动军政军民团结融合深度发展，驻鹤解放军、武警部队和民兵预备役为地方发展作出了重要贡献。民族宗教和外事侨务、对台、统计、档案、史志、地震等工作取得新成绩。

　　各位代表，在经济下行压力加大、结构性矛盾突出等情况下，我市经济社会平稳较快发展，保持了良好的趋势、态势和气势，对外美誉度越来越高，干部职工思想行动统一度越来越高，整体发展协调度越来越高，人民群众满意度越来越高。这是省委、省政府和市委正确领导的结果，是市人大和市政协大力支持的结果，是全市人民拼搏奉献的结果。在此，我代表市人民政府，向广大工人、农民、知识分子、干部、解放军指战员、武警官兵、公安民警以及社会各界人士致以崇高的敬意，向关心支持我市现代化建设的朋友们表示诚挚的感谢！

　　在总结成绩的同时，我们也清醒地认识到经济社会发展中还存在一些突出矛盾和问题：一是经济总量偏小、产业结构不优、发展层次不高的问题依然存在，转型提升发展任务艰巨；二是部分行业、企业生产经营困难，要素保障从紧，经济指标保持较快增长压力增大；三是城乡基础设施和公共服务设施相对薄弱，公共产品和公共服务欠账较多，老城区和农村建设发展仍有不少困难；四是政府职能转变、简政放权不够到位，一些公务员思想观念、能力素质、工作作风与形势要求和人民期盼还不适应，服务环境还需要持续优化。对此，我们一定高度重视，认真对待，采取切实措施加以解决，绝不辜负人民期望。

　　二、2015年工作总体要求和主要目标

　　今年是全面深化改革的关键之年，是全面推进依法治国的开局之年，是全面完成“十二五”规划的收官之年，也是为率先全面建成小康社会奠定坚实基础的重要一年。当前，我市与全国、全省一样，经济发展进入新常态，面临的宏观大势、外部局势、自身态势都发生了深刻变化。整体上看，我市仍然处于可以积极进取、主动作为的战略机遇期，同时也处于社会转型的矛盾多发期和爬坡过坎、升级突破的关键期。我们既要保持清醒、趋利避害、迎难而上，更要坚定信心、抢抓机遇、赢得先机，千方百计化挑战为机遇、化机遇为优势，牢牢把握发展的主动权。

　　今年政府工作总体要求是：全面贯彻落实省委、省政府和市委的决策部署，主动适应新常态，积极融入全省现代化建设大格局，坚持提升求进工作总基调，注重发挥优势、突出重点、先行先试、统筹协调，力争在转型发展、农业现代化、新型城镇化、生态宜居、社会治理创新上示范先行，努力打造最优发展环境和最优从政环境，促进经济平稳健康发展和社会大局和谐稳定，加快生态活力幸福之城建设进程，迈出率先全面建成小康社会新步伐，为中原更加出彩作出应有贡献。

　　经济社会发展主要目标是：在优化结构、提高质量、改善环境基础上，生产总值增长9%以上，全社会固定资产投资增长18%以上，规模以上工业增加值增长11.5%左右，一般公共预算收入增长11%左右，城镇居民人均可支配收入、农民人均纯收入均增长9%以上，社会消费品零售总额增长12%左右，居民消费价格涨幅控制在3%左右，城镇新增就业3万人以上，城镇化率提高1.5个百分点左右，节能减排完成国家节能减排财政政策综合示范市要求目标，环境质量改善完成省下达任务。

　　贯彻总体要求，实现预期目标，需要全面贯彻市委八届七次全会和市委经济工作会议精神，准确把握生态活力幸福之城的深刻内涵、“提升求进”总基调的核心要义和整体布局的重大关系，做到一个适应，主动适应发展速度变化、经济结构优化、增长动力转换的新常态，用现代化思维和改革创新精神深入研究把握重大问题，善于在变化中捕捉机遇，努力用改革办法、市场手段和开放途径推进科学发展提升发展。着力两个融入，深度融入国家粮食生产核心区、中原经济区、郑州航空港经济综合实验区三大国家战略规划和国家“一带一路”建设，主动融入全省全面建成小康社会、加快现代化建设格局，打造开放合作新高地，争当示范先行排头兵。突出三个坚持，坚持把深化改革作为推动发展的根本动力，坚持把扩大开放作为加快发展的综合举措，坚持把创新驱动作为转型发展的重要引擎，推动经济增长从要素投入为主向改革推动、开放带动、创新驱动转换。处理好四个关系，处理好稳定增长与提质增效的关系，坚定不移把发展作为第一要务，以大质量引领发展全局，加快产业结构、城乡结构、要素结构优化升级，及时防范化解风险，把经济社会发展推向质量时代；处理好强化传统优势与培育新型优势的关系，巩固提升我市区位交通、产业发展、现代农业、生态环境、政务服务等传统优势，努力培育竞争新优势，持续做精品、创品牌、成特色；处理好经济发展与改善民生的关系，准确把握经济社会发展与人民生活改善的结合点，把稳增长与惠民生相统一，把扩大内需与实施民生工程相结合，以经济发展增进人民福祉，以改善民生构筑经济增长的长期动力；处理好当前发展与着眼未来的关系，以勇于担当的责任感和只争朝夕的紧迫感夯实发展基础，以更加开阔的眼界和示范先行的追求科学谋划未来五年发展，引领全市人民为加快建设生态活力幸福之城、率先全面建成小康社会而不懈努力。

　　三、2015年重点工作

　　（一）保持经济平稳较快增长。坚持项目建设更加突出重点，招商引资更加突出集群，企业服务更加突出实效，要素保障、载体平台更加完善到位，促进经济持续健康运行。一是持续提升三项重点工作水平。项目建设方面，认真开展“项目质量年”活动，以“促投资、抓进度、保成效”为核心，创新投资方式，注重投资效益，全面提高项目开工率、资金到位率、投产达效率，力争150个市重点项目完成投资400亿元以上、新谋划100个以上大项目好项目。招商引资方面，整合招商力量，创新招商方式，强化企业主体地位，突出产业招商、专业招商、以商招商、精准招商，注重招大引强、招新引高，注重引项目引资金与引平台引智力相结合，落实完善招商引资奖励政策、招商项目跨县区摆放“飞地”政策等，组织承办好首届快餐食品博览会、第六届镁博会等系列特色招商活动，着力抓好30个拟落地、30个签约和40个重点在谈项目的跟踪落实，力争引进省外资金260亿元、境外资金7.3亿美元，进出口总额增长8%。企业服务方面，牢固树立服务企业就是服务发展的理念，将“扶优”与“救急”、“渡难关”与“促转型”结合起来，重点扶持一批带动作用强、成长性高的企业，帮助开展用工对接、产销对接等，提高服务企业的针对性和实效性。二是不断增强三项要素保障能力。金融服务方面，积极争取省级金融机构在信贷规模、业务授权、创新试点等方面给予支持，保持货币信贷平稳增长；有效开展银企对接活动，搭建金融机构与重点项目、中小微企业对接平台；优化程序并扩大“助保贷”业务量；设立过桥资金池，防止资金链断裂带来风险；充分利用资本市场，探索建立文化旅游、金属镁、汽车产业发展基金和股权投资基金，鼓励支持更多企业通过境内外上市、新三板挂牌、发行私募债等方式直接融资。建设用地方面，推进国土空间优化发展实验区建设，优化建设用地布局；认真执行土地利用规划，依法规范和集约节约使用土地，挖潜土地使用潜力，盘活现有存量土地，着力解决批而未征、征而未供、供而未用问题，稳妥推进新建房地产和城市综合体类商业地产项目，提高土地供地率和投资强度；做好城乡建设用地增减挂钩、人地挂钩和农村土地综合治理工作，保障发展用地。人力资源方面，实施全民技能振兴等系列专项提升行动计划，完成职业技能培训3万人次以上，创新服务企业用工模式，改善人力资源供给结构；着力培育引进一批高层次急需人才，用好本土人才，充分发挥好院士工作站、博士后科研工作站的作用，实实在在解决好人才的后顾之忧，使人才引得来、留得住、用得好。三是继续夯实三个载体平台。推动产业集聚区提质转型创新发展，不断强主导、改体制、晋位次，着力完善供气、供热、污水处理等基础设施和检验检测、技术创新等公共服务设施，力争浚县、鹤淇、金山3个产业集聚区达到一星级标准，宝山创建二星级产业集聚区，提升国家经济技术开发区、省高新技术产业开发区、省经济开发区发展水平。加快服务业“两区”建设，完善实施好“两区”建设专项推进方案和专项规划，打造淇水湾文化旅游风情街、商业步行街等一批特色商业街区，建设一批适合服务业融合发展的城市综合体、商务楼宇等新型载体，推进40个超亿元项目建设，力争完成投资50亿元以上，不断提升“两区”的集聚辐射能力。完善用好口岸平台，拓展完善海关和出入境检验检疫局功能，加快推进镁加工、电子信息等省级出口基地建设，积极申报综合保税区、汽车电子电器特色产业基地，努力打造全省一流、辐射豫北的区域性开放平台。

　　（二）加快产业转型升级。实施好主导先导产业集群培育行动计划，加快培育2个千亿级和6个超百亿级产业集群，着力提升全国质量强市示范城市建设水平，推动制造向创造转变、速度向质量转变、产品向品牌转变。一是改造提升传统主导产业。坚持发挥优势、延链补链、对接终端，加快推进煤基新能源新材料基地建设，积极推进煤化一体化项目，建成投产鹤淇电厂和可降解塑料等5个精细化工项目，实施好华石能源新型煤化工项目二期、鹤煤集团低热值煤发电等项目，力争煤电化材一体化产业实现主营业务收入800亿元；围绕打造中国快餐和早餐食品工业化基地，加快推进赛德50万吨快餐、超汇工业园等18个总投资144亿元的重点项目，年内完成投资41亿元，持续提升“鹤壁食品”影响力，力争食品产业实现主营业务收入600亿元；加快宇风改装车、天海国际高端线束出口工业园等12个总投资82亿元的在建重点项目建设，积极推进汽车整车项目，完成投资20亿元以上，力争汽车零部件产业实现主营业务收入90亿元。二是做大做强新兴先导产业。坚持扩规模、成集群、提效益，加快推进特色电子信息产业基地和“中国镁谷”建设，努力把新兴产业培育成主导产业。落实完善电子信息产业发展政策，实施好华塑高分子新材料产业园、荣兴电子科技产业园等总投资105亿元的11个在建重点项目，服务好上海斐讯通信产业基地等总投资119亿元的6个新开工项目，力争电子信息产业实现主营业务收入60亿元；完善提升国家镁及镁合金质检中心，加快建设镁交易中心、富士康科技园，壮大镁业集团，力争金属镁精深加工产业实现主营业务收入80亿元。同时，积极抓好聆海家居、朝歌纺织等高成长型企业发展，力争把现代家居、纺织服装2个产业打造成新的经济增长点。三是提速发展现代服务业。突出休闲旅游和新型物流两个新业态，在特色化、品牌化上下功夫求实效。休闲旅游方面，围绕项目建设、配套服务、体制创新三个突破提升，以“一区两带四板块”为重点，推进旅游综合体、浚县古城、古灵山、淇河生态文化旅游度假区、淇水湾朝歌文化园、华漫文化旅游城、古瓷窑博物馆等10个文化旅游重点项目建设，发展壮大文化旅游集团，积极推进旅游资源整合和链条衔接，探索全域旅游体制改革新模式，创建国家生态旅游示范区；挖掘提升淇河文化、鬼谷子文化、民俗文化、春节文化等，叫响“诗意淇河·生态鹤壁”品牌，实现接待游客、旅游总收入分别增长15%和20%以上。新型物流方面，大力发展第三方、第四方物流，鼓励制造企业分离物流业务，争取年内新增3家3A级物流企业；完善物流信息服务、煤炭现货交易、电商物流交易等新型物流平台，搭建物流企业与郑州航空港、保税物流园区、郑欧班列等对接推介平台，加快煤炭物流、商贸物流、食品物流等专业园区建设，开工建设晋能集团、省投资集团2个煤炭物流基地，积极创建省级示范物流园区；以电子商务为切入点，加快培育壮大农业硅谷产业园、宏昌物流园、店连店电商科技园等重点企业，促进有形市场与无形市场、线上线下融合发展，引领新型物流跨越发展，力争物流业增加值占第三产业增加值的10%以上。

　　（三）提升现代农业水平。瞄准全国前列目标不动摇，以创建国家现代农业示范区为抓手，加快推进粮食生产方式、农业经营体系、物资装备、农业产业体系、农业信息科技现代化，持续提高农业综合效益和竞争力。一是不断壮大农业产业集群。全面提升3个省级示范产业集群发展水平，力争形成销售收入超10亿元的农业产业集群10个；提升发展22个市级以上现代农业产业园区，加快鹤浚快速通道都市生态农业带、城乡一体化示范区都市生态农业园等建设。二是积极完善农业经营体系。积极培育新型农业经营主体，引导规范土地经营权有序流转，新发展农民专业合作社100家以上、家庭农场200家以上；积极创建全国农业社会化服务综合标准化示范市，提升农业综合标准化服务水平；培养一批高素质职业农民，激发农民的创新、创业活力。三是切实增强物资装备能力。落实农业政策，加大资金投入，推进农田水利灌溉、引水补源和病险水库除险加固等工程建设，持续夯实农业发展基础；总结推广高产创建模式，完成42个小麦和39个玉米万亩高产示范片创建任务，新建高标准粮田16万亩；优化农机装备结构，提升农业机械化水平；加快推进气象现代化建设，积极创建全国现代农业气象示范市，增强防灾减灾和稳定增收能力。四是持续强化科技信息支撑。加快农业科技创新和重大技术推广，推进良种育繁推一体化，建成投用11个基层农技推广区域站，力争农业科技贡献率达到60%以上；完善农业信息化综合管理服务平台，实施好农业信息进村入户工作，健全农业信息化服务体系。五是加快转变农业发展方式。减少农业投入品过量使用，逐步退出超过资源环境承载能力的生产，推进农业废弃物转化利用，严格保护耕地和水资源，建设资源节约、环境友好农业。

　　（四）科学推进新型城镇化。突出农业转移人口市民化、土地制度改革和城乡一体化示范区、中心镇、美丽乡村建设，进一步提高新型城镇化建设质量和管理水平。一是重点提升单元组合型城市建设水平。高标准完善城乡总体规划、淇河右岸控制性详细规划以及城市道路、供热等专项规划。突出淇水湾商务休闲区、高铁广场城市综合体、电子新城核心区等重点区域建设，淇水湾商务休闲区6个主骨架项目主体完工、6个配套项目形成施工高潮；提升淇滨区建成区功能，提高商务中心区和中心城区辐射带动能力。统筹提升老城区建设发展，进一步完善基础设施和公共服务设施，老城区供热覆盖面积达到350万平方米，持续改善生活生产环境。推进两县新老城区联动协调发展，壮大县域、镇域经济，增强综合承载能力和发展活力。二是着力统筹城乡一体化发展。深化城乡一体化试点工作，落实完善“1+10”政策体系，促进人口有序转移；积极稳妥推进新农村和新型社区建设，抓好太行山东麓和淇河沿岸美丽乡村示范带建设；启动第二批100个农村人居环境示范村创建，保护宣传好古村落，持续改善农村人居环境。三是继续完善城乡基础设施和公共服务设施。加快建立多元化城镇建设筹资机制，统筹实施好鹤淇电厂中水回用和集中供热等一批城镇基础设施扩能增效项目，建设一批公共服务设施；加快现代综合交通体系建设，着力推进鹤辉高速等总投资64亿元的10个重点交通项目，年内完成投资25亿元，鹤浚快速通道、东大线年内主体完工，新老区快速通道改建启动，县乡村公路新建220公里。四是切实加强城市精细化管理。扎实做好国家智慧城市试点工作，利用好鹤壁城市云计算中心建设成果，完善公共信息平台、数字城管平台功能；改革创新城市管理服务体制机制，加强和改进交通秩序、社区物业、卫生死角、城市噪音等治理，加快天赉渠、二支渠、汤河等城市河道治理和背街小巷路面改造，积极创建国家卫生城市和国家园林城市，推进城市自然绿化、精细绿化、立体绿化，提升美化净化绿化及管护水平；出台政策措施，制定完善规划，多方筹资投入，更新公交车辆200台，新增公共自行车2000辆，切实方便居民出行。

　　（五）全面深化改革创新。继续统筹推进、勇于攻坚，努力在若干领域取得实质性进展，加快构建有利于科学发展的体制机制。一是扎实推进各项改革。支持鹤山区综合改革实验区加快发展，支持山城区资源型城市转型发展，支持开发区、宝山产业集聚区、城乡一体化示范区提升发展。全面完成市县政府机构改革，稳步推进事业单位分类改革。深化财税金融体制改革，完善预算管理机制，推进部门预决算公开，改革财政科技经费分配机制，清理规范财税优惠政策；推进金融机构改革重组，启动农村金融改革试点工作。深化农村综合改革，稳步开展农村土地承包经营权、宅基地使用权确权颁证和集体林权、人地挂钩改革，积极发展“土地银行”和“农村土地信托”。统筹推进医药卫生体制、产业集聚区和服务业“两区”建设管理体制机制、交通运输体制机制、商事制度、公车制度、价格、行政区划等改革。二是着力提升科技创新能力。积极培育创新主体、搭建创新平台、完善创新载体、实施创新专项，积极推动科技开放合作，促进以科技创新为重点的全面创新。充分发挥财政资金的引导作用，积极创建科技金融试点市；引导企业加大技术创新投入，提高企业自主创新能力。深化市校市院合作，建立完善产业研发平台和科技金融创新创业服务平台，充分发挥中科院半导体研究所河南研究院、国家镁合金材料工程技术研究中心鹤壁中心、南京林业大学河南聆海家居研究院等平台作用，发挥省肉禽和市高性能镁及镁合金、汽车电器、光电子等产业技术创新战略联盟作用，积极推进中科院上海有机化学研究所鹤壁研究院、中国农科院大用肉禽研究院等研发机构建设，多出科技创新成果，多将成果就地转化。三是大力发展民营经济。落实完善鼓励支持民营经济发展的政策措施，实施转型升级引领企业培育工程和民营企业、中小企业健康发展行动计划，引导中小微企业突出特色和主业，增强产品竞争力，培育一批“小巨人”企业。充分尊重企业家的创造、选择、个性，引导支持企业家改革创新、外引内联，不断把企业做大做优做强。

　　（六）推进文化发展繁荣。扎实推进精神文明建设和文化事业、文化产业发展，提高城市的文化内涵和品位，为人民群众提供更多高质量的文化服务。一是深入推进文明创建。积极开展中国特色社会主义和中国梦宣传教育，深入推进社会主义核心价值观体系建设，深化群众性精神文明创建，大力弘扬鹤壁英模精神，持续开展“鹤壁好人”评选活动，进一步倡树文明新风尚；加快推进社会信用体系建设，提高全社会的诚信意识和信用水平。二是加快发展文化事业。健全公共文化服务体系，继续实施好文化活动进基层等文化惠民工程，加强乡镇综合文化服务中心建设，提高文化中心和艺术中心等利用率开放度；加大文艺优秀人才培养引进力度，激励调动各方面的积极性创造性，推出更多具有鲜活时代气息、浓郁地方特色的精品力作；做好卫河（永济渠）浚县段、黎阳仓遗址、大赉店遗址、鹤壁古瓷窑等文化遗产保护工作。三是培育壮大文化产业。充分发挥牛派艺术、陶瓷、泥塑、石雕手工艺等文化资源优势，努力打造文化产业集群；支持四季青文化广场等做大做强，引导企业提供更多个性化的文化产品和服务，不断提高文化消费水平。四是着力打造节会品牌。结合我市文化旅游资源优势，打造常态化的节会活动和文化演出，有序举办民俗文化节、樱花节、诗歌会赛、文博会等，持续办好省歌舞演艺集团月演出、“芝麻官大舞台”周演出以及1000场日常文化演出，提高层次和内涵，丰富群众文化生活。

　　（七）打造生态宜居优势。围绕创建国家园林城市、国家卫生城市和建设国家可持续发展实验区，统筹推进生态环境保护、生态产业发展和生态文化建设，加快绿色低碳循环发展，努力让美丽与发展同行。一是巩固提升绿色发展。扩大国家森林城市创建成果，持续推进以“三廊三带三区”为骨架、覆盖全域的林业生态体系建设，完成造林绿化19.8万亩。加大淇河保护立法力度，加快推进淇河国家湿地公园和盘石头水库生态环境保护、淇河流域环境治理及生态保护等项目，让群众享受到更多的“绿色福利”。二是重点推进低碳发展。扎实做好国家节能减排财政政策综合示范市、中美生态低碳试点城市工作，强力推进重点领域节能减排，大力发展节能环保朝阳产业，投资15亿元推进产业园区改造提升、绿色建筑行动、绿色低碳交通、可再生能源利用等示范工程。坚持源头严防、过程严管、后果严惩，实施好蓝天、碧水和乡村清洁等工程，着力提升大气、水等环境质量。落实好节能产品惠民政策，优化用好宝贵的环境容量资源，倡导绿色消费理念和生活方式，让鹤壁的天更蓝、水更清、地更绿。三是持续加快循环发展。围绕创建国家循环经济示范市，重点抓好宝山产业集聚区循环化改造、山城区陶瓷园区工业废弃物综合利用示范基地建设，推进大宗固体废弃物和城镇生活废弃物资源化利用，工业固体废弃物利用率达到95%以上。完善循环型产业体系，促进产业间、企业间相互衔接，推动循环经济由提供产品向提供技术、标准、服务转变。

　　（八）持续保障和改善民生。坚持把为民便民、高效优质作为社会治理的出发点和落脚点，集中力量把省市重点民生工程办实、办快、办好，提升民生发展质量，维护社会公平正义。一是努力扩大就业增加收入。完善就业创业服务体系，健全基层服务平台功能，发挥创业带动就业的积极作用，重点做好高校毕业生、城镇困难人员、退役军人、残疾人等重点人群的就业工作，继续提高劳动力就近就地就业比例，新增农村劳动力转移就业2万人。落实好强农惠农富农政策和失业保险稳岗补贴政策，健全职工工资正常增长机制和支付保障机制，农村和社区干部工资标准人均每月分别提高200元、400元。力争完成8个贫困村整村推进扶贫开发工作。全面完成盘石头水库第三期移民搬迁安置工作。二是持续提升社会保障水平。确保保障性安居工程质量进度，提高分配效率，新开工995套、基本建成8131套、分配安置5000套；完成农村危房改造1500户。扩大社会保障覆盖面，新农合和城镇居民医保财政补助标准提高到380元，基本公共卫生服务经费财政补助标准提高到40元，实施城镇居民大病医疗保险；提高企业退休职工养老金、城乡低保对象补助水平、优抚对象抚恤金和生活补助标准，60岁以上城乡居民参保人员基础养老保险金人均财政补助标准提高18元，实现城乡居民与城镇职工养老保险转移接续，推进机关事业单位养老保险制度改革，新增发放社会保障卡20万张；完善城乡社会救助体系，做好“救急难”和高龄津贴发放工作，保障困难弱势特殊群体基本生活。三是大力发展社会事业。实施好第二期学前教育三年行动计划，推进义务教育均衡发展、普通高中内涵式发展，统筹民办教育和特殊教育协调发展，持续解决学前教育入园难、义务教育大班额等难题，加快市示范性综合实践基地建设，新建和改扩建学校7所、主体完工或建成投用学校3所，改善农村地区、贫困地区办学条件和师资队伍，确保80%成建制的农村中小学实现优质资源班班通；大力发展现代职业教育，从秋季起对全日制中职在校有正式学籍学生全免学费，提高鹤壁职业技术学院、汽车工程职业学院、能源化工职业学院等办学质量，建设优势特色专业，通过校校合作实现本科院校零的突破。进一步整合用好医疗卫生资源，加强基层医疗人才培养，推进城市社区卫生服务中心等建设，建成投用市人民医院主病房楼并做好三甲医院创建工作，深化与北京中医药大学、上海东方肝胆病外科医院、河南省肿瘤医院等合作，提升医疗服务水平，努力实现患者就近就地就医；认真落实计划生育政策，促进人口长期均衡发展。加快建设全民健身活动中心暨体育公园，启动市体育中心建设，广泛开展全民健身运动，打造环淇河自行车赛等知名体育赛事。做好养老服务业综合改革试点工作，完善养老服务体系，健全老年福利制度，加快城市社区养老服务中心、城乡养老服务设施建设，努力实现老有所依、老有所养、老有所乐。解决1.76万名农村居民和农村学校师生的饮水安全问题。提升慈善、残疾人、妇女儿童、红十字等事业发展水平。四是切实加强和创新社会治理。牢固树立“大平安”理念，持续改进社会治理方式，引导社会组织有序参与社会治理，加快从传统社会管理向现代社会治理转变。加强基层基础工作，健全基层公共服务平台，及时反映和协调解决群众利益诉求。完善社会矛盾预防化解机制，落实依法逐级走访制度，积极化解土地征用、征地拆迁、环境污染治理等矛盾问题，依法打击非法集资等违法行为。严格安全生产、环境保护、食品药品等监管，加强和改进应急管理。规范市场秩序，打击侵权假冒行为。健全立体化社会治安防控体系，不断提升平安建设水平，提升群众安全感、满意度和幸福感。

　　加强国防教育和国防后备力量建设，支持驻鹤解放军、武警部队、民兵预备役和人民防空建设，建成投用武警鹤壁支队机动大队和市消防支队训练基地项目，深入开展双拥共建，促进军政军民团结融合深度发展。支持工会、共青团、妇联、科协、工商联等人民团体发挥桥梁纽带作用，注重发挥商会、社团等社会组织的作用。积极做好民族宗教和外事侨务、对台、统计、档案、史志、地震等工作。

　　科学编制“十三五”规划，广泛吸收人大代表、政协委员和社会各界意见，加强政策衔接和项目对接，争取更多重大项目、重要试点、重点工作纳入省和国家规划，努力培育后续竞争力。

　　四、加强政府自身建设

　　围绕打造最优发展环境、最优从政环境的目标，以建设法治政府、廉洁政府和服务型政府为重点，深入推进“改作风、转职能、优环境”活动，不断提高政府执行力、公信力和服务水平。

　　加快转变政府职能。坚持发挥市场配置资源的决定性作用和更好发挥政府作用，深化行政审批制度改革，持续减少审批事项，优化审批程序，加大非行政许可审批事项清理力度，全面清理规范社会中介机构和行政事业性收费、经营服务性收费；加强行政审批事中事后监管，切实做到“放”、“管”结合，提升行政审批标准化管理水平；更好地协调发挥市、县区行政服务中心的作用，推进一口受理、限时办理、规范办理、透明办理和网上办理，为企业、群众提供更加便捷优质的服务。

　　深入推进依法行政。严守法治原则，一切依法办事，做到“法无授权不可为、法定职责必须为”。完善重大行政决策法定程序，强化对行政权力的制约和监督，坚决执行人大及其常委会的决议决定，支持政协参政议政，自觉接受人大法律和工作监督、政协民主监督，认真办理人大代表建议和政协提案，加强行政、司法、监察、审计等监督，广泛听取民主党派、工商联、无党派人士和人民团体的意见。深化政务公开，充分发挥电子政务网等平台作用，对涉及群众利益的事做到公开透明、公正公平，让权力在阳光下运行。

　　持续改进工作作风。纠正“四风”和树立新风并举，推动作风建设常态化长效化。坚持以人为本，从群众最急迫的事情做起，从群众最关切、最敏感的地方改进，做到对群众深恶痛绝的事零容忍、对群众急需急盼的事零懈怠。坚持担当有为，该做的事即使有压力有难度也要做，该担的责挺起腰杆也要担，该管的事即使得罪人也要管。坚持务实重干，把更多的精力投入到科学谋划、狠抓落实上，持续提升发展意识、发展水平、工作标准、工作举措和工作实效，实现全市发展和人民福祉全方位、高质量的进步。

　　加强反腐倡廉建设。严格落实廉政建设责任制，从严执行“一岗双责”，把廉政建设要求融入到政府工作各个环节。严明政治纪律，严守政治规矩，严格正风肃纪，严肃执纪问责。全面落实中央八项规定，牢固树立过紧日子思想，厉行节约，反对浪费，严格规范财政资金管理使用，珍惜和用好每一分财政资金。加强对政府系统工作人员的严格教育、严格监督、严格管理，始终保持清廉本色，做人民满意的公务员。

　　各位代表！面对经济社会发展的新常态新任务，我们深感责任重大、使命光荣。让我们把思想和行动统一到省委、省政府和市委的决策部署上来，以争先晋位、示范先行的追求，以改革创新、勇于担当的精神，以紧盯不放、一抓到底的韧劲，凝神聚力，务实重干，奋发有为，全面完成各项目标任务，加快生态活力幸福之城建设进程，迈出率先全面建成小康社会新步伐，为中原更加出彩作出应有的贡献！
